# Microparticles with tunable, cell-like properties for quantitative acoustic mechanophenotyping

**DOI:** 10.1038/s41378-023-00556-6

**Published:** 2023-07-12

**Authors:** Ryan Dubay, Eric M. Darling, Jason Fiering

**Affiliations:** 1 Center for Biomedical Engineering, Brown University, Providence, RI 02912 USA; 2 Biological Microsystems, Draper, Cambridge, MA 02139 USA; 3 Department of Pathology and Laboratory Medicine, Brown University, Providence, RI 02912 USA; 4 School of Engineering, Brown University, Providence, RI 02912 USA; 5 Department of Orthopaedics, Brown University, Providence, RI 02912 USA

**Keywords:** Physics, Engineering, Materials science

## Abstract

Mechanical properties of biological cells have been shown to correlate with their biomolecular state and function, and therefore methods to measure these properties at scale are of interest. Emerging microfluidic technologies can measure the mechanical properties of cells at rates over 20,000 cells/s, which is more than four orders of magnitude faster than conventional instrumentation. However, precise and repeatable means to calibrate and test these new tools remain lacking, since cells themselves are by nature variable. Commonly, microfluidic tools use rigid polymer microspheres for calibration because they are widely available in cell-similar sizes, but conventional microspheres do not fully capture the physiological range of other mechanical properties that are equally important to device function (e.g., elastic modulus and density). Here, we present for the first time development of monodisperse polyacrylamide microparticles with both tunable elasticity and tunable density. Using these size, elasticity, and density tunable particles, we characterized a custom acoustic microfluidic device that makes single-cell measurements of mechanical properties. We then applied the approach to measure the distribution of the acoustic properties within samples of human leukocytes and showed that the system successfully discriminates lymphocytes from other leukocytes. This initial demonstration shows how the tunable microparticles with properties within the physiologically relevant range can be used in conjunction with microfluidic devices for efficient high-throughput measurements of mechanical properties at single-cell resolution.

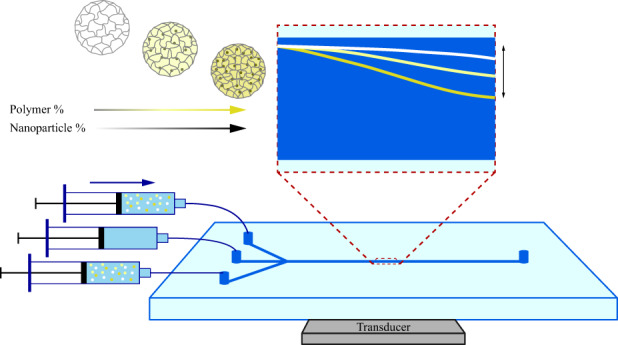

## Introduction

Cellular mechanical properties, i.e., mechanophenotype, have been shown to correlate with biomolecular state. Specifically, differences in cellular mechanophenotype have been associated with cancer malignancy^1^, leukocyte activation^2^, stem cell pluripotency and differentiation potential^3^, and cytoskeletal and nuclear response to drugs^4^. Detectable shifts in cell mechanophenotype provide an alternative label-free approach for routine monitoring of the dynamic cell states. This monitoring is particularly of interest when assessing therapeutic efficacy in high-throughput drug screening, stem cell differentiation progress and associated quality controls, trauma or infection screening indicated by leukocyte activation, and transplant rejection^4^. Unfortunately, existing methods such as atomic force microscopy^5^, micropipette aspiration^6^, microbead rheometry^7^, optical tweezers and traps^8^ are comparatively time-consuming and not well suited for real-time, continuous monitoring of large cell populations. Label-free flow-based deformation microdevices^9^ are promising alternatives to conventional methods as they can measure cell mechanical properties at rates more than four orders of magnitude greater than conventional methods^10^. One such emerging technique is microfluidic acoustophoresis, which is already widely used for sorting, purification, and cell positioning in cytometry. In acoustophoresis, a cell’s ultrasonically induced displacement at known acoustic conditions can be compared to a known function of its size, compressibility, and density^11,12^.

Careful calibration of these novel microdevices is required in order to make reliable measurements of cell mechanophenotype and to enable their translation to commercial use. Currently, rigid microbeads of polymers such as polystyrene (PS) are used for calibration because they can be purchased in a variety of sizes, surface chemistries, and fluorescence with polydispersity indexes less than 2%, making them ideal for studying size dependent behavior in flow. However, the elastic modulus of PS is ~3 GPa^13^, which is roughly six orders of magnitude greater than that of living cells (~0.1 to 10 kPa)^14^. This raises the concern that conventional microparticles, which are constrained to their intrinsic mechanical properties, are insufficient for calibrating and characterizing microdevices that measure multiple cellular mechanical properties beyond size alone (e.g., density, and compressibility). It should be noted, biological cells, for example cell lines, are also unsatisfactory candidates for calibration particles as genetic and phenotypic drift is observed during serial passaging^15^ and primary cells are inherently variable and nonuniform. Hence, a stable particle of tunable size, compressibility, and density is needed for calibration in clinical and industrial settings.

Toward this objective, we previously developed hydrogel microparticles (MPs) with tunable elasticity comparable to that of cells. The MPs are monodisperse, in part thanks to fabrication by a flow-focusing droplet generator, which is itself a microfluidic device but unrelated to the cell measurement devices. We showed that polyacrylamide MPs can be used to calibrate a simple microfluidic device containing a constriction segment to quantify the elastic modulus of cells^16^. Having calibration particles of well-defined size and elastic modulus allowed for the experimental derivation of a generalized equation connecting these parameters to the inertially-driven particle migration due to the constriction.

**Fig. 1:**
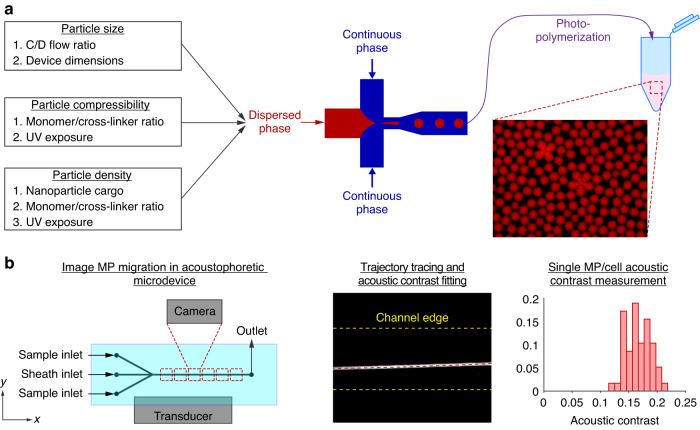
High-level study overview. **a** Design parameters used for the fabrication of size, elastic modulus, and density tunable MPs. Diagram of MP fabrication process within a flow-focusing microfluidic droplet generator, previously described^16^, and subsequent photopolymerization. **b** Illustration of acoustophoresis microdevice with example image of particle trajectory and automated trajectory trace, shown as red dashed line. Output data from MP lot as histogram of acoustic contrast measured using acoustophoretic method

In the current study, we improve our capabilities to replicate the mechanical phenotype of cells by introducing density modulation via nanoparticles embedded in the MPs, while preserving the ability to tune both size and elastic modulus independently. We fabricated MP lots with varying size, polyacrylamide composition, and nanoparticle loading and measured their mechanical properties using two independent methods, conventional instrumentation and an acoustophoretic microdevice. We show that both methods provide similar results for MP acoustic properties, but that the acoustophoretic enables measurement of individual MPs to provide insight into the population heterogeneity in a high-throughput manner (Fig. 1). Having characterized and validated the acoustophoretic microdevice with tunable MPs, we used the MPs to test if the acoustophoretic method could identify subpopulations of MPs within a mixed sample having MPs of varied acoustic properties but similar size, a test which is not possible using PS beads. Finally, we applied the MP calibration and used the label-free acoustophoresis technique to identify the percentage of a leukocyte subtype, lymphocytes, within leukocytes from three human donor fresh blood samples. Having MPs in the relevant cell-like range of density and compressibility allowed the appropriate device settings to be predicted in advance for the leukocyte measurements.

## Results

### Production and characterization of mechanically tunable MPs

**Fig. 2:**
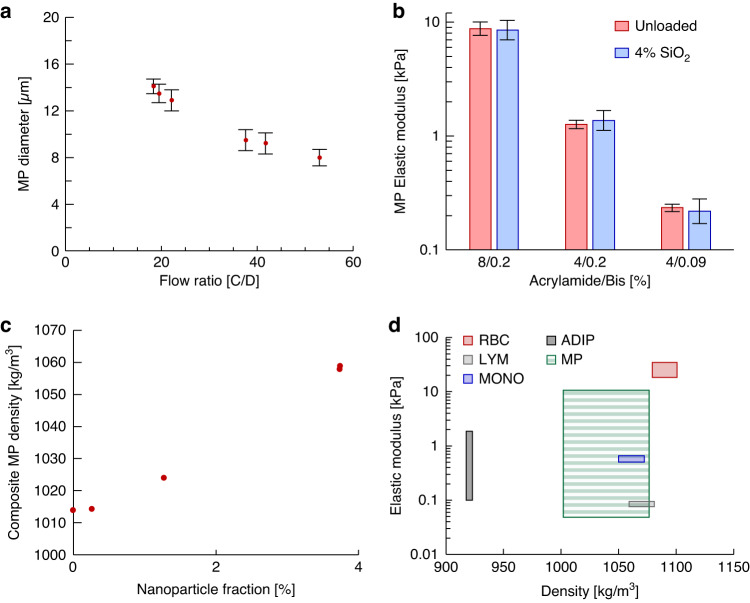
Representative results of MP fabrication. **a** Unloaded (i.e., no nanoparticle loading) MP diameter (±standard deviation) as a function of the continuous-to-dispersed (C/D) phase flow rate ratio. **b** MP elastic modulus is controlled via monomer (acrylamide) and crosslinker (bis-acrylamide) concentrations. **c** Composite MP density, when suspended in deionized water, as a function of % SiO_2_ nanoparticle loading. **d** Ranges of density and elastic modulus for selected cell types and for the tunable MPs (MP) used in this study. Selected cell types are red blood cells (RBC)^17,18^, lymphocytes (LYM)^17,19^, monocytes (MONO)^19,20^, and adipocytes (ADIP)^5,21^

To fabricate monodisperse MPs we used a microfluidic flow-focusing droplet generator of rigid and inert materials as previously described^16^. MP size was controlled by adjusting the continuous-to-dispersed phase flow rate ratios within the droplet generator, where increasing this ratio resulted in smaller MPs. Particle elastic modulus was controlled by the ratio of acrylamide monomer to bis-acrylamide crosslinker, where increasing the monomer and crosslinker percentages increased the MP elastic modulus^16^. The MP density was independently modulated by the volumetric percent of SiO_2_ nanoparticles (NP) suspended in the hydrogel precursor solution. Increasing the volume fraction of SiO_2_ resulted in an increase in the composite MP density. Note that the composite MP density comprises collectively the polyacrylamide (PAM) matrix, hydrating suspension fluid (e.g., deionized water), and optional embedded NPs. The composite density was calculated by measuring the density of the MP suspension of known particle concentration and average particle size and comparing this to the measured density of the suspending fluid alone. Inclusion of SiO_2_ nanoparticles did not affect the elastic modulus of the MPs. These results are collectively shown in Fig. 2.

MPs were suspended in deionized water and their size, density, and speed of sound were measured by conventional instruments as described in section “Microparticle characterization in bulk suspension”. Size measurements were required to calculate the MP volume fraction in suspension measurements of density and speed of sound as well as to calculate the acoustic contrast in the acoustophoretic method. When taken together, MP density and speed of sound relative to the suspending fluid allows for the calculation of the MP acoustic contrast.

The specific microfluidic droplet generator design used in this study produces polyacrylamide MPs with diameters ranging from ~9 to ~28 μm while maintaining a polydispersity index (PDI) below 10%^16^. The diameter of the MP lots fabricated for this study ranged from 11.0–16.4 μm, density from 1003–1068 kg/m^3^, and speed of sound from 1502–1543 m/s. The achievable density range for this study was governed by the selected NP cargo (SiO_2_) and polyacrylamide formulations required for fabricating MPs of physiologically relevant elastic moduli. To minimize aggregation of NPs in the precursor solution during fabrication, we restricted the NP volume fraction to ≤4%, which limited the composite MP density to ≤1070 kg/m^3^. Using gold instead of SiO_2_, we anticipate the composite MP density range to exceed 1200 kg/m^3^. Using the bulk suspension measurements, a total of six MP lots, each having different characteristics within these ranges, were selected for acoustophoretic characterization.

To measure the MP acoustic contrast by acoustophoretic displacement, the device was first calibrated as follows. PS beads were pumped through the microchannel at varying flow rate and transducer voltage, and images of the resulting trajectories were analyzed to determine their lateral (*y*) displacement vs. downstream position. The downstream position was converted to time, using the calculated local velocities from the known volumetric flow rate. The acoustic energy density, *E*_ac_, was calculated by fitting the observed transverse position *y*(*t*) to an established model^22^ for one-dimensional acoustophoretic displacement, Eq. (1)

1 $$y(t)=\frac{1}{{k}_{y}}\,{{\mbox{arctan}}}\,\left\{\,{{\mbox{tan}}}\,\left[{k}_{y}y(0)\right]\,{{\mbox{exp}}}\,\left[\frac{4{{\Phi }}}{9\eta }{\left({k}_{y}a\right)}^{2}{E}_{ac}t\right]\right\}$$

where *y*(0) is the transverse position at *t* = 0, *k*_*y*_ is the wave number defined as *π* divided by the channel width (${k}_{y}=\frac{\pi }{{c}_{w}}$), Φ is the acoustic contrast factor, *η* is the dynamic viscosity, and *a* is particle radius. In this equation, the acoustic contrast is defined as

2 $${{\Phi }}=\frac{5\gamma -2}{2\gamma +1}-\frac{1}{\gamma {\beta }^{2}}$$

where *γ* and *β* are defined as the density and speed of sound ratios of the particle relative to the suspending fluid, respectively. For the PS calibration microbeads, we used previously reported values of 1050 kg/m^3^ for the density and 1700 m/s for the speed of sound^22^. The density and speed of sound of the suspending fluid (0.05% Tween 20 in water) was measured experimentally at 997 kg/m^3^ and 1499.2 m/s, respectively.

**Fig. 3:**
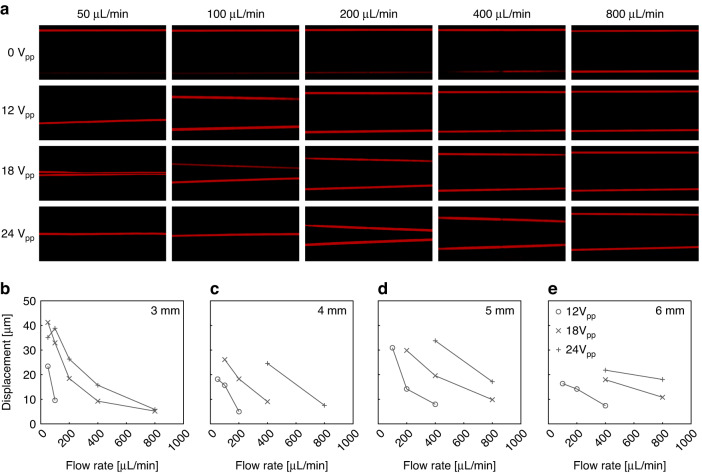
Experimental parameters (i.e., flow rate and driving voltage) impact focusing of MP in acoustophoretic microdevice. **a** Representative false colored micrographs of MP (*a* = 13.4 μm, Φ ≈ 0.15) acoustophoretic migration acquired 4 mm away from inlet at the five flow rates and four voltage conditions tested. Acoustically driven MP displacement for the MP lot shown in A across the experimental conditions at **b** 3 mm, **c** 4 mm, **d** 5 mm, and **e** 6 mm away from the inlet trifurcation. Displacement values were discarded if less than a minimum net migration of >4 μm

Following this calibration, diluted PAM MPs were infused into the acoustophoretic microdevice. Fluorescent trajectories were imaged in 1 mm segments starting at 2 mm and ending at 7 mm from the inlet trifurcation. Combinations of total flow rate and driving voltage were systematically tested at each of the six discrete locations, with the flow rates and voltages shown in Fig. 3. Unsurprisingly, the average MP displacement increased as driving voltage increased and the average displacement decreased as the flow rate increased (Fig. 3).

Using the calculated *E*_*ac*_ from the prior PS-bead calibration for the specific driving voltage and longitudinal location, MP acoustic contrast (Φ) was calculated by fitting the PAM MP transverse position and time data to Eq. (1)^22^. As a confirmation of the approach, we note that no statistical correlations were observed between driving voltage, flow rate, longitudinal position, or MP displacement and the resulting acoustic contrast calculation (Supplementary Fig. S6).

**Fig. 4:**
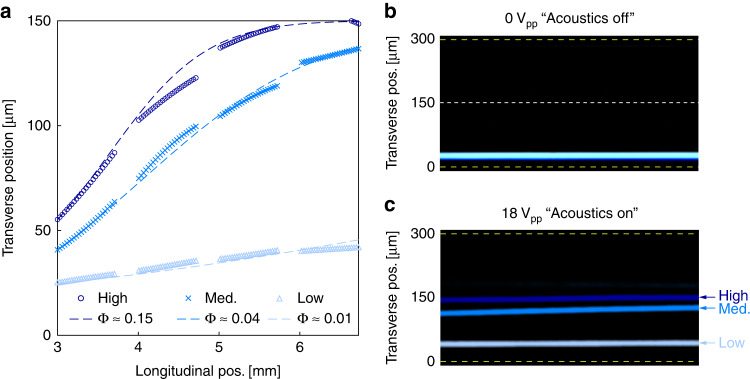
Whole chip mapping of MP trajectories. **a** Mapped trajectories for MPs of three different formulations with presumed differing acoustic contrast, “High”, “Med.”, and “Low”. Dashed lines indicate calculated ideal trajectories using the corresponding average calculated value of Φ. Composite false colored images of the three MP lots shown in part a at the 5 mm longitudinal position for **b** acoustics off and **c** 18 V_pp_ conditions. Dashed yellow lines indicate channel edges and channel centerline is represented by the dashed white line at 150 μm transverse position

Images of PAM MP fluorescent trajectories were acquired at each of the six different longitudinal locations (image field of view) during the initial MP characterization. This permitted greater dynamic range and precision in measuring the MP acoustic contrast. The optimum measurement is made between the extremes, away from the channel wall (i.e., 0 μm) and away from the terminal position located at channel centerline (i.e., 150 μm). High contrast particles traversed this range further upstream than low contrast particles. For example, under similar experimental conditions, MPs of 13.4 μm in diameter and ~0.15 acoustic contrast reached their terminal transverse location near the 5 mm longitudinal position, while MPs of 16.4 μm in diameter and ~0.04 acoustic contrast still exhibited acoustophoretic migration beyond 6 mm. This range is shown in Fig. 4, which overlays actual trajectories of the differing MPs at each of the longitudinal locations. Additionally, the figure shows idealized trajectories for the respective MPs, which were calculated using Eq. (1) with the average measured MP radius, an average of the acoustic contrast values determined from each longitudinal location, and the calibrated acoustic energy density converted to a continuous function of longitudinal position (Supplementary Fig. S5). Hence, imaging at several longitudinal locations permitted us to measure the MP acoustic contrast across a wide range (e.g. Φ ≈ 0.01–0.15).

**Fig. 5:**
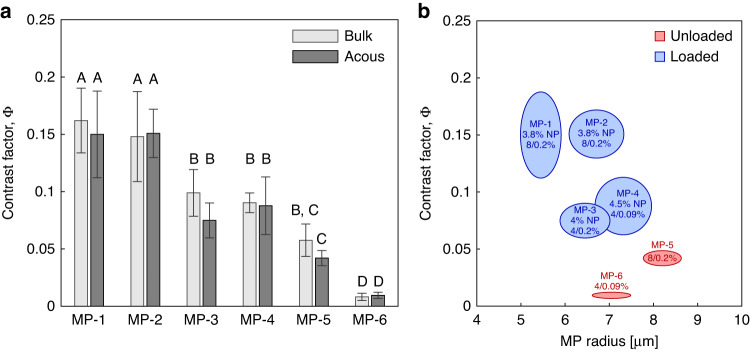
Characterization of the MP lots used in study. **a** Measured acoustic contrast factor of each of the lots, where light and dark gray bars represent values obtained via bulk suspension or acoustophoretic methods, respectively. Statistical pairwise comparisons are indicated by letters “A”–“D”. **b** MP radius and contrast factor values of the same lots, where the width and height of the ellipse corresponds to the standard deviation for that attribute. The nanoparticle volume fraction used is indicated by X% NP while the monomer and crosslinker percentage is represented as shown in Fig. 2b. Blue and red ellipses correspond to MPs with and without SiO_2_ nanoparticles, respectively

Having mapped the fluorescent trajectories of the six MP lots and subsequently calculated their acoustic contrast, we compared these values of Φ acquired via acoustophoretic methods with those obtained in bulk suspension using the conventional instruments. The two methods were in agreement within statistical significance (p-value < 0.01). These comparisons are shown in Fig. 5a, where MPs of statistically similar acoustic contrast share the same letter. MP lots 1 and 2 (i.e., MP-1 and MP-2) exhibited the greatest acoustic contrast, while MP-6 was the lowest. Notably, the acoustophoretic method identified four unique statistical groups of acoustic contrast, while the bulk methods only identified three, suggesting that the former method has greater resolution.

The mean radius and acoustic contrast for the six particle lots tested are shown in Fig. 5b, where the width and height of the ellipse correspond to the radius and acoustic contrast standard deviations. Additionally, the formulations are reported as percent NP volume fraction (X%) and percent of acrylamide and bis-acrylamide (acrylamide/bis-acrylamide%). Based on the composite formulations, we would expect to observe four unique levels of acoustic contrast, as successfully identified using the acoustophoretic method.

### Identification of distinct lots from mixed MP suspensions

**Fig. 6:**
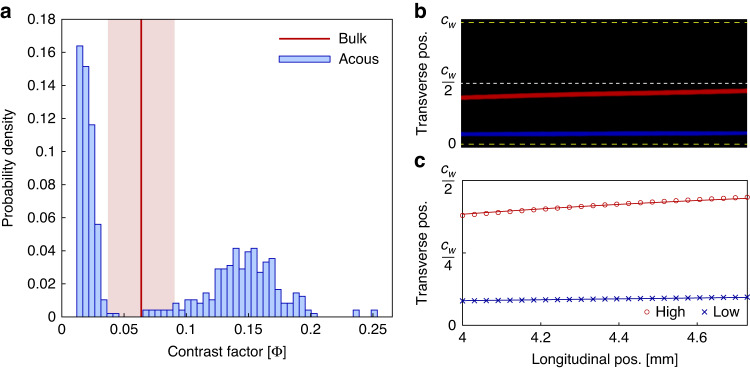
Measurement of acoustic contrast for a mixture of equal parts of “High” (~0.15) and “Low” (~0.01) acoustic contrast MPs, of similar diameter (~13.4 μm). **a** Histogram of individual MP contrast factors measured via the acoustophoretic method, along with the red line and shaded region representing the average and standard deviation of the contrast factor measured using bulk suspension methods. **b** Representative false colored image of two particle trajectories, where red and blue trajectories correspond to “High” and “Low” contrast factor MPs, respectively. **c** Traced trajectories for image shown in part b, with the ideal trajectory represented by the solid line

Once we confirmed the two quantification methods resulted in similar measurements of MP acoustic contrast, we compared the feasibility of identifying two distinct lots within a mixed sample by the two characterization methods. Samples from two MP lots with similar diameters, 13.4 *μ*m, but different acoustic contrasts, ~0.15 and ~0.01 (i.e., lots MP-2 and MP-6), were mixed together in equal quantities, and the suspension was analyzed by the microdevice. Using a combination of dilute MP suspension and long exposure times relative to MP residence time in the field of view, we were able to record long but non-congruent trajectories, where each trajectory corresponds to a single particle. Figure 6 shows the resulting distribution in measured contrast factor along with representative trajectories. For this purpose, we developed a computational algorithm to analyze the distribution of measured particle displacements and determine if a bimodal or normal distribution fit the data better (Supplementary Fig. S7). Also shown is the mean measurement of the bulk mixed suspension using the conventional instruments. Of the 482 MP trajectories analyzed, the algorithm classified 242 (i.e., 50.2%) as low acoustic contrast while the remaining 49.8% were of high contrast. The average of the acoustophoretic data was calculated (0.083 ± 0.066), which was not statistically different from the bulk measurement of the mixed sample (0.064 ± 0.014). This finding emphasizes the shortcomings of bulk measurements when used on heterogeneous samples.

The average acoustic contrast of the high contrast population was ~0.151 when measured on its own and ~0.146 for the mixed sample. The average acoustic contrast of the low contrast population was ~0.009 when measured individually but ~0.019 for the mixed sample experiment. The disagreement between the two experiments may be explained by the overall lower displacement of the low contrast MPs observed during the mixed experiment compared to the unmixed experiment. The high contrast MPs used in the mixed study experience an acoustic force that is ~15× greater than the low contrast MPs. As such, the experimental conditions required to observe acoustically driven migration within the optimal range resulted in a median displacement closer to the lower limit of detection compared to the unmixed experiment (i.e., lower signal-to-noise).

### Quantification of human leukocyte subpopulation percentages

Given the successful identification of two MP populations within an ideal test sample, we next applied the methodology and calibration to use the acoustophoresis device to identify cellular subpopulations within a complex biological sample. In this case the acoustic contrast and therefore the resulting displacement among the cell types is expected to differ, even when size differences are accounted for. White blood cells with a non-specific fluorescent stain, purified from fresh whole blood from three human donors, were passed through the acoustic microdevice and their trajectories were imaged 5 mm away from the inlet trifurcation. Aliquots from the same samples were also analyzed by gold standard methods of automated hematology analyzer or flow cytometry.

**Fig. 7:**
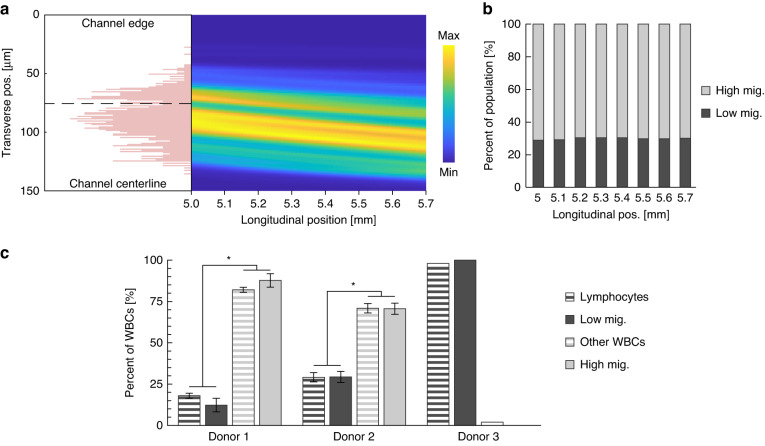
Acoustic characterization of white blood cell population. **a** Representative heat map of white blood cell trajectories for Donor 2 whole blood sample, with histogram of cell position at the upstream end and corresponding bimodal cut point designated by dashed line. Channel edge and centerline correspond to transverse position values of 0 and 150 μm, respectively. **b** Percentage of high- and low-migration leukocytes relative to longitudinal location of sampling. **c** Pairwise comparison of subpopulations (low- and high migration) measured by the acoustophoretic method (striped) and the lymphocyte population measured by conventional cell analyzers (solid)

Figure 7 shows the results of the white blood cell analysis. Two populations were consistently identified within the distribution of observed displacements, termed low- and high migration. As a further test of the robustness of the method, varying the longitudinal location where the displacements were sampled did not affect the final measured percentages of low- and high-migration cells. In each donor, the percentage of low-migration cells was in agreement with the lymphocyte percentage (p-value ≪ 0.01), as measured by conventional means. The lymphocyte population in the first two samples was measured by hematology analyzer. The third donor sample contained only lymphocytes (~98%), specifically natural killer (NK) cells, which are a subtype of lymphocytes. These had been isolated just prior to the experiment using an immunomagnetic negative isolation kit. In this third sample, as expected, analysis of the trajectories identified only a single population of low-migration cells.

## Discussion

The major focus of this study was to fabricate and characterize MPs with tunable acoustic properties for use as a calibration particle in acoustic microdevices or other microfluidic devices that are sensitive to cell density and compressibility. Since these MPs exhibit acoustic properties within physiologically relevant ranges, we were able to use them to select optimal experimental conditions for the subsequent cell measurements. Without such particles, two unsatisfactory approaches would have to be adopted to allow polystyrene microspheres to better represent acoustophoretic cell trajectories: (1) increase acoustic impedance of suspending fluid or (2) use a microparticle of smaller size. Fluid additives that increase the acoustic impedance tend to also strongly increase viscosity of the suspending fluid, and this can in turn introduce artifacts that are not relevant to ordinary media in which cells would be analyzed. Additionally high viscosity decreases throughput due to the increased stokes drag on the particle’s cross-flow migration, and increases the acoustic boundary layer which can introduce acoustic streaming phenomenon^23,24^. Alternatively, to compensate by reduced particle size, PS microspheres need to be on the order of <2 μm in radius to replicate acoustically driven migration of a typical lymphocyte suspended in phosphate-buffered saline. Particles at that size can experience a secondary force induced by acoustic streaming, which could introduce artifacts^23^.

In analyzing six lots of MPs (Fig. 5), each fabricated under different conditions, the acoustophoretic method successfully identified the four unique groups of acoustic contrast from the MP lots tested, while the bulk method identified only three. Based on the predetermined formulations, when accounting for both hydrogel composition and NP loading, we anticipated four statistically different groups of acoustic contrast, with the other two varying only in size. Specifically, MP-1 and MP-2 were fabricated of the same polyacrylamide formulation and nanoparticle volume fraction. Both MP-3 and MP-4 comprised the same acrylamide concentration but MP-3 contained ~2× more crosslinker than MP-4. The 0.5% greater SiO_2_ nanoparticle volume fraction in MP-4 offsets this difference in polyacrylamide composition between the two lots by bringing the difference in predicted composite density <1%. Both MP-5 and MP-6 were fabricated without SiO_2_ nanoparticles but MP-5 contained twice as much monomer and crosslinker. The failure of the bulk method to discriminate the four unique lots suggests that the acoustophoretic method has superior resolution between the two methods.

Both measurement techniques are subject to error, where 9.7% and 3.3% relative standard error were observed for the bulk and acoustophoretic methods, respectively. In particular, the bulk suspension method suffers from inaccuracies in the volume fraction of MPs in suspension and temperature inaccuracies during speed of sound acquisition. By our estimates, these two main sources of error accounted for >90% of the total measurement error present. Alternatively, the acoustophoretic method permits a greater number of replicates from the same quantity of cells, reducing the impact of spurious outliers, which is an inherent limitation for the bulk suspension technique. Similar to bulk suspension methods though, error in MP diameter accounts for approximately 50% of the error in the calculated acoustic contrast. Image alignment and rotation corrections were incorporated into the Python-based analysis software to reduce displacement measurement error, which was anticipated to contribute ~35% in calculated acoustic contrast error.

While the acoustophoretic method addresses some shortcomings of the bulk methods, limitations are still present within the current system. Specifically, an average cell size (diameter or volume) in a population must be measured by other means and applied in order to calculate the acoustic contrast from a given particle or cell’s trajectory. Although not difficult to measure via additional instrumentation (e.g., Coulter counter or optical microscopy), instantaneous measurements of size for each particle of interest would improve the accuracy of the calculated acoustic contrast. Improved optics and imaging could permit simultaneous measurement of particle size along with trajectory mapping. Additionally, the design of the microfluidic flow cell and acoustic actuation could be better optimized to allow for a more uniform acoustic energy density in the longitudinal direction of the chip, which in these studies was averaged over the ~728 μm field of view.

In the white blood cell analysis, we compared the percentages of low- and high-migration cells to the populations measured using an automated hematology analyzer (Sysmex), which counts three leukocyte subpopulations based on size using the Coulter principle: lymphocytes, neutrophils, and a remainder group consisting of monocytes, eosinophils, and basophils. The acoustic radiation force is dependent on particle volume, where larger cells experience greater acoustic migration than smaller cells of similar contrast. Since lymphocytes are the smaller leukocyte subtype, we compared them to the low-migration group observed in our device, and we compared the other two leukocyte subtypes as a combined group ("other WBCs") to the high-migration group (Fig. 7). As expected, the percentage of low-migration cells were statistically similar to the lymphocyte percentage and the high-migration cell percentages were similar to the Other WBCs percentages. Interestingly, the average NK acoustic contrast was statistically greater than the low-migration cells from the two white blood cell experiments, when assuming the same size for all lymphocyte subtypes. This agrees with recently reported results on acoustic migration of lymphocyte subpopulations, where NK cells experienced greater acoustic mobility compared to T and B lymphocytes^25^.

Although the acoustophoretic method was unable to discern all three leukocyte subtypes recognized by the hematology analyzer, it has other advantages in cell analysis applications where differences in cell compressibility and density are of particular interest. For example, cytoskeletal changes that correlate with biomolecular states (e.g., malignancy^1,12^) can alter the compressibility or density of cells. We believe the inability of the acoustophoretic method to discriminate the neutrophil group from the monocyte/eosinophil/basophil group occurs because acoustic-driven migration convolves both size (i.e., volume) and acoustic contrast, and here the properties offset each other. In contrast, the label-free hematology analyzer used in this study classifies leukocyte subtypes based solely on their measured volume but provides no information on density or compressibility. As discussed above, we envision enhancements to the acoustic device that could simultaneously measure cell size.

**Fig. 8:**
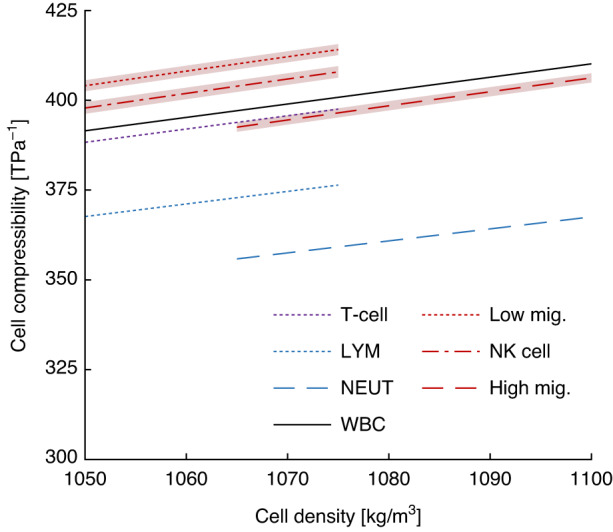
Calculation of cell compressibility using measured/reported acoustic contrast and assumed typical cell densities. Data obtained during this study is shown in red, while other values in blue and black were taken from literature: Dabbi et al. reported the compressibility of T-cells^27^, Augustsson et al. reported the acoustic impedance of lymphocytes (LYM) and neutrophils (NEUT)^11^, and Cushing et al. reported the compressibility of WBCs^26^

Using the acoustic contrast measured here combined with known density ranges for the white blood cell subtypes, their corresponding adiabatic compressibility can be calculated and compared with other published values. The calculated compressibility for the three leukocyte subpopulations identified, low-migration, high-migration, and NK cells are shown in red in Fig. 8. The values agree with values measured by Cushing et al.^26^ through bulk suspension methods, where only a single value for all white blood cells (from a whole blood sample) was reported. Interestingly, when taking a weighted average of low- and high-migration cell compressibility we obtain a value that differs <0.1% to the WBC value by Cushing et al.^26^. Dabbi et al. incrementally increased the density of an isotonic suspension media to observe the crossover point where T lymphocytes exhibit negative contrast (i.e., migrate away from pressure nodes) in an acoustophoretic microdevice, and by that method obtained a compressibility of 392 TPa^−1^, which differs only <3% from our value for low-migration cells^27^. Augustsson et al. measured the acoustic impedance of lymphocytes and neutrophils by monitoring their terminal acoustic focusing position within a microchannel having a stable acoustic impedance gradient that increased towards the channel centerline. Similar to this study, the lymphocytes exhibited greater compressibility than the neutrophils, but the values are offset from our measurements^11^. One explanation for the discrepancy may be differences in temperature between the two studies.

Others have previously demonstrated cell characterization using acoustophoresis in microchannels. Augustsson et al. measured the acoustic contrast of single cells (BA-F3, MCF7, human monocytes, lymphocytes, and neutrophils) with an acoustic impedance gradient that increased towards the channel centerline^11^. This method has the benefit that it is size independent as it relies on balancing the acoustic impedance of cells to that of the suspending fluid. However, throughput was limited to ~11 cells/s, and also required precise control and characterization of the flow field. Fu et al. measured the acoustic-driven trajectory in a microchannel, similar to that used here, testing A549 cells with and without epithelial-mesenchymal transition induction^12^. Image-based measurement of cell sizes appeared to limit throughput, which was estimated to be ~0.3 cells/s based on limited information provided. Garofalo et al. calculated the compressibility of leukocytes using density values reported earlier^26^ along with the measured acoustic separation of a mixed suspension of reference polystyrene microparticles and cells^28^. Bogatyr et al. measured the size, density, and compressibility of individual microparticles and two cell lines by tracking gravitational settling in addition to acoustophoresis within a static acoustic chamber^29^. Similarly, Bellebon et al. measured the gravitation settling and acoustophoretic migration of individual mesenchymal stromal cells to measure their size, density, and compressibility within a closed acoustic cavity^30^. While this method provides quantitative measurements of all three cell properties and is useful for studying temporal cellular physical alterations, it suffers from low throughput, ~1.3 cells/s^29^ and ~1 cell/min^30^, due to settling times and low cell concentration within the field of view to allow for tracking of individual cells; moreover, it is not in continuous flow, limiting its utility for larger samples. In comparison to these studies, this work achieved a throughput of >500 cells/s in continuous flow with a single low viscosity fluid; however it has the drawback that assumptions of average size limited the accuracy. Additionally, similar to studies by Augustsson et al., Fu et al., and Garofalo et al., it does not deconvolve the contributions of density and compressibility to the measurement of acoustic contrast.

This study demonstrated for the first time the fabrication of microparticles with independently tunable size, elastic modulus, and density. These MPs of tunable mechanical properties permitted the characterization and identification of operating parameters for the study of biological samples. Since the MP mechanical properties closely resembled that of the biological samples, we were able to perform preliminary screening of operating parameters similar to those used for studying single-cell trajectories of leukocyte subtypes. We anticipate many additional applications could benefit from the use of MPs with tunable mechanical properties for improving cell analysis and separation devices.

## Methods

### Microparticle production

Fluorescent polyacrylamide MPs were fabricated as previously described^16^, with additional details outlined in the Supplementary Information. Briefly, PAM precursor solution and 2.5% Hypermer SP6 (Croda International) in 1-octadecene (technical grade, 90%, MilliporeSigma) served as the dispersed and continuous phases, respectively. Both solutions were infused into a custom microfluidic droplet generator via syringe pump (Harvard Apparatus Pump 33 DDS, Harvard Apparatus). The continuous-to-dispersed phase flow rate was adjusted to achieve desired MP diameter (Fig. 2), while MP elastic modulus and density were determined by the precursor formulation. For low crosslinker concentrations in ideal network polyacrylamide gels, increasing the concentration of bis-acrylamide crosslinker without changing the amount of acrylamide monomer in the precursor solution resulted in MPs of greater elastic modulus^31^. To increase the density of MPs, deionized water (DI) was supplemented for an aqueous suspension of SiO_2_ nanoparticles. MPs were cured within a 100 mm long borosilicate glass capillary tube (1.0 mm inner diameter, 1.2 mm outer diameter, VitroCom) affixed to the droplet generator polytetrafluoroethylene outlet tubing. Two 365 nm LEDs, with collimating and focusing lenses, were positioned above the capillary tube such that the object plane was positioned before the focal plane to produce a ~6 mm long illumination region so the PAM precursor droplets resided in the illumination region for greater than five seconds at >61.9 mW/cm^2 ^^32^. The photopolymerized MPs were collected in 1.5 mL conical tubes and allowed to rest for at least three days, followed by three washes with 0.1% Triton X-100 (MilliporeSigma) and three washes with DI water, after which they were resuspended in 1 mL of DI water.

### Microparticle characterization in bulk suspension

After washing, MPs were allowed to equilibrate in DI water for at least 24 h. MP size was then measured by microscopy and image analysis in MATLAB^Ⓡ^.

MP concentrations were measured using an automated cell counting instrument (Cellometer^Ⓡ^ Vision, Nexcelom Bioscience), for controlled sample volume and phase contrast imaging, along with a custom MATLAB^Ⓡ^ counting script. Counts were performed in replicates of four to reduce error. Once average MP concentration was acquired, MP volume fraction was calculated by random sampling of previously measured MP diameters from normal distribution, with skewness and kurtosis information, or random sampling with replacement of MP diameters that were not normally distributed.

A digital density meter (DMA 1001, Anton Paar) was used for the bulk suspension measurements. Five to seven MP suspensions with known volume fraction, including particle-free suspension fluid which served as 0% volume fraction, were measured at 26 °C. MP density was calculated by extrapolating the suspension density versus MP volume fraction linear fit to 100% volume fraction.

Similarly, the MP speed of sound was calculated by measuring the speed of sound of suspensions of known MP volume fraction at 26 °C using an acoustic microscope (Sonoscan Gen6, Nordson Text & Inspection). The microscope records the time interval between incidence of 20 MHz pulse upon the top surface of the suspension (at the fluid-solid interface within a custom test cell) and the reflection of the pulse as it again arrives at this surface. The test cell of polyetherimide (PEI) was fabricated to reduce sample volume required to 40 μL. Measurement of this time interval was first recorded for water, and all suspension measurements were scaled according to an assumed value of 1499.6 m/s for the speed of sound in water at 26 °C^33^. Each volume fraction sample was prepared to allow for eight infusions into the test cell, with five replicate measurements taken in 30 second intervals. Multiple infusions were performed to reduce error caused by inhomogeneities in MP dispersion within the suspension. The test cell was submerged in a water bath with thermocouple and heating element connected to a temperature controller (Model 250, J-KEM Scientific Inc.). A secondary thermocouple was mounted to the side of the flow cell with thermal paste and silicone RTV adhesive (DOWSIL^TM^ 3140, Dow) such that the probe was approximately 600 μm from the fluid cavity to provide instantaneous temperature measurement of the fluid being measured. Suspension speed of sound was corrected for temperature differences across the experiment using a standard curve for water sound speed at different temperatures. Once suspension sound speed was measured, the MP sound speed was calculated using Wood’s equation^34^.

Following the density and speed of sound measurements, the average MP acoustic contrast was calculated using Eq. (2). The relative uncertainty of the calculated acoustic contrast was then calculated using the law of propagation of uncertainty.

### Microparticle acoustophoretic characterization

A 3-inlet Y-channel glass flow cell with microchannel cross section 300 × 100 μm, (Translume Inc.) was bonded to a 10 × 21 × 0.8 mm type 840 piezoelectric transducer (APC International) with cyanoacrylate adhesive (LOCTITE^*Ⓡ*^ 401). The acoustic microdevice was mounted to a temperature controlled stage as previously described^35^. The transducer was driven with a 2.34 MHz sine wave produced by an arbitrary function generator (AFG3022C, Tektronix Inc.) that was passed to an RF amplifier (AG1020, T&C Power Conversion Inc.). Peak-to-peak voltage, current, and RMS power were measured via a voltage probe (TPP0100, Tektronix Inc.), current probe (P6022, Tektronix Inc.), and calculated by an oscilloscope (DPO2004B, Tektronix Inc.), respectively. At least 30 images of particle streamlines were captured at 7 frames per second for each experimental condition using a CCD camera (Retiga R6, Teledyne Photometrics) mounted to a microscope (Axio Zoom/V16 ZEISS). A camera exposure time of 80 ms was used for flow rates ≤100 μL/min, and 40 ms otherwise. The parameters of particle concentration, volume of fluid in the field of view, exposure time, and flow rate were adjusted to achieve a range of 1–8 trajectories per image and to ensure that the probability of two particles following the exact same trajectory is negligible. Similar to bulk suspension methods, acoustophoretic measurements were performed at 26 °C.

PS and PAM MP suspensions were diluted to 8 × 10^4^ mL^−1^ in 0.05% Tween 20 to mitigate surface adhesion to flow cell walls. Samples were infused through the side inlets such that MPs were hydrodynamically positioned near channel walls prior to acoustic migration towards the channel centerline, while 0.05% Tween 20 sheath fluid was passed through the center inlet at a 20/80 ratio, respectively. Polyvinylidene fluoride coated magnetic stir discs (5 mm diameter and 1.7 mm thick, V *&* P Scientific, Inc.) were placed inside the sample syringes and were continuously agitated by a rotary magnetic tumble stirrer (V *&* P Scientific, Inc.) to prevent settling within the syringe.

To calibrate the average acoustic energy density at locations throughout the microchannel, acoustic migration of fluorescent 7.32 μm PS microspheres (Bangs Laboratories Inc.) were measured for the four driving voltages and at least two flow rates (Fig. 3) at 2–7 mm away from the inlet trifurcation in 1 mm increments. For each condition, at least 30 images were acquired for post-processing. Similarly, PAM MPs were then passed through the acoustic microdevice and trajectories were imaged for each condition shown in Fig. 3.

### Cell preparation and acoustophoretic characterization

Normal healthy human whole blood and leukapheresis product, both anticoagulated with acid citrate dextrose were procured from Research Blood Components, LLC and Charles River Laboratories Inc., respectively. All blood products were received de-identified from the vendors. Initial complete blood counts were measured for each sample using an automated hematology analyzer (XP-300, Sysmex Corporation).

For the unpurified leukocytes, whole blood samples were washed with 1X phosphate-buffered saline (PBS, Gibco^TM^) to remove platelets followed by red blood cell lysis using 10X RBC Lysis Buffer (BioLegend, Inc.). Remaining white blood cells (WBCs) were stained with carboxyfluorescein succinimidyl ester (CellTrace^TM^ CFSE, Invitrogen^TM^) at a final 5 μM concentration. A complete blood count was again performed after which the stained WBCs were washed and resuspended at 1 × 10^5^ mL^−1^ in 0.2 μm filtered 1X PBS. Alternatively, for the NK cell sample, red blood cells were removed from leukapheresis product via Ficoll density medium centrifugation (Ficoll-Paque^TM^ PLUS, Cytiva). NK cells were then isolated from the buffy coat fraction using immunomagnetic negative isolation (EasySep^TM^ Human NK Cell Isolation Kit, STEMCELL Technologies Inc.) and purity was measured via flow cytometry by gating from all viable single cells (Attune^TM^ NxT, Life Technologies). Isolated NK cell concentration and viability were measured using an imaging cytometer (Celigo Image Cytometer, Nexcelom Bioscience LLC) from an acridine orange and propidium iodide stained aliquot. Similar to WBCs, the isolated NKs were then stained with CFSE and suspended in CTS^TM^ NK-Xpander^TM^ Medium (Gibco^TM^) at 1 × 10^5^ mL^−1^.

Similar to methods described in section “Microparticle acoustophoretic characterization”, *E*_*ac*_ was calibrated for at least three flow rates for each of the driving voltages at 5 mm away from the inlet trifurcation. Once the device was calibrated, fluorescently stained cells were passed through the side inlets while the sheath fluid comprised of cell suspending fluid was infused through the center inlet. Cell suspensions within the syringe were continuously agitated similarly to PS and PAM MPs. Images of fluorescent cell trajectories were acquired at 5 mm away from the inlet trifurcation. Approximately 10 mL of sample was passed through the acoustic device for each experiment.

### Image processing and analysis

Fluorescent trajectory images were processed using custom Python-based GUI software, with additional information found in the Supplementary Information. Typical processing steps included removing image slices that contained no or partial particle trajectories from the tiff stack, followed by suitable contrast enhancements (e.g., linear and sigmoidal), z-stack projection, and background removal. Processed images were exported in their native file format.

From the processed fluorescent trajectory images, the MP longitudinal and transverse positions were calculated. The elapsed time was then calculated using transverse position dependent instantaneous velocity that was interpolated from the 2-dimensional steady state Poiseuille velocity field for the given flow rate^36^. The MP was assumed to be in the mid-plane relative to the channel height for all elapsed time calculations. Acoustic energy density and acoustic contrast factor values were calculated from transverse positioning and elapsed time data using Eq. (1) for 1-dimensional transverse position^22^, for PS and PAM particles, respectively.

Calculated contrast factors for each experiment were statistically assessed in R. Zero voltage (i.e., acoustics off) and conditions resulting in <4 μm total particle migration were removed from analysis. An analysis of variance (ANOVA) was performed to compare calculated acoustic contrast factor values and quantification methods. Games-Howell pairwise comparisons were performed to establish statistical groupings.

To assess the presence of subpopulations within the sample for the PAM MP bimodal and biological cell studies, we used a Tukey Lambda probability plot correlation coefficient (PPCC) to identify the shape parameter that best describes the initial and final position data sets. Initial and final positions were defined as the transverse MP or cell position at the leftmost and rightmost longitudinal locations within the fluorescent micrograph. The optimal cutoff, which separates the bimodal distribution into two separate distributions above and below, was identified by systematically separating the sorted data at cutoff values between the 1 and 99% quantiles. The strictly standardized mean difference was then calculated between the two separated groups for each cutoff value. The optimal cutoff was identified as the value resulting in the maximum prominence of the strictly standardized mean difference (Supplementary Fig. S8)^37^.

## Supplementary information

Microparticles with tunable, cell-like properties for quantitative acoustic mechanophenotyping supplementary information
