# Supplementary material for: Microparticles with tunable, cell-like properties for quantitative acoustic mechanophenotyping
Source: Microsyst Nanoeng. 2023 Jul 12;9:90. doi: 10.1038/s41378-023-00556-6 (PMC10336031; doi:10.1038/s41378-023-00556-6)
Supplement: Supplementary file 1 — Microparticles with tunable, cell-like properties for quantitative acoustic mechanophenotyping supplementary information [file 41378_2023_556_MOESM1_ESM.pdf]

Microparticles with tunable, cell-like properties for quantitative acoustic mechanophenotyping

R. Dubay[1,2], E. M. Darling[1,3,4,5], J. Fiering[2]

[1] Center for Biomedical Engineering, Brown University, Providence, 02912, RI, USA
[2] Biological Microsystems, Draper, Cambridge, 02139, MA, USA
[3] Department of Pathology and Laboratory Medicine, Brown University, Providence, 02912, RI, USA
[4] School of Engineering, Brown University, Providence, 02912, RI, USA
[5] Department of Orthopaedics, Brown University, Providence, 02912, RI, USA

S1. Microparticle fabrication

Polyacrylamide-based microparticles (MP) were fabricated using a microfluidic flow-focusing droplet generator containing a 40 µm nozzle as previously reported [1]. Polyacrylamide precursor solution served as the dispersed phase, while 1-octadecene with 2.5% Hypermer SP6 served as the continuous phase. The polyacrylamide precursor solution was composed of acrylamide (40% Acrylamide Solution, Bio-Rad Laboratories, Inc.), bis-acrylamide (2% Bis-Acrylamide Solution, Bio-Rad Laboratories, Inc.), ammonium persulfate (Bio-Rad Laboratories, Inc.), lithium phenyl-2,4,6-trimethylbenzoylphosphinate (LAP) photoinitiator (≥95%, MilliporeSigma), fluorescent dye (Sharpie), and deionized (DI) water. All MP formulations contained 8% fluorescent dye, 0.1% (w/v) ammonium persulfate, and 0.1% LAP photoinitiator. For MP formulations containing nanoparticles (NP), the DI water component was replaced with NP suspensions. All SiO_2 NP suspensions were purchased at 50 mg/mL and concentrated to ~10% volume fraction in Milli-Q water (100 nm hydroxyl-terminated SiO_2 NPs, Alpha Nanotech Inc.). MP formulations and microfluidic droplet generator operational parameters are shown in Table S1.

Table S1. Polyacrylamide precursor formulations for the six MP lots tested in this study, with Lot ID corresponding to IDs found on Fig. 5. “Disp.” and “Cont.” correspond to dispersed and continuous phase, respectively. “Fab.” corresponds to fabrication, i.e., droplet generation rate calculated by dividing dispersed phase flow rate by average precursor droplet volume.

Lot ID	% Acryl	% Bis	% SiO_2	Disp. Flow [µL/min]	Cont. Flow [µL/min]	Fab. Rate [kHz]
MP-1	7.67%	0.20%	3.75%	2.00	80.00	131.7
MP-2	7.68%	0.20%	3.75%	2.40	90.00	84.8
MP-3	4.17%	0.20%	4.63%	2.25	80.00	88.1
MP-4	3.97%	0.09%	4.72%	2.05	87.00	83.8
MP-5	7.67%	0.20%	0%	3.60	68.00	55.9
MP-6	3.97%	0.08%	0%	2.25	85.00	102.3

S2. Python-based GUI

Once images of fluorescent particle/cell trajectories were acquired, the 16-bit TIF images were read into the custom Python-based software for image processing, trajectory tracing, acoustic contrast or acoustic energy density calculations, and data concatenation. The typical processing pipeline started by loading the image stack into the software from the “File” tab, along with the preestablished 2D velocity matrix spreadsheet (Fig. S1), which was calculated using previously reported equations [2].

Next, in the “Image Transform” tab, image slices containing no complete (i.e., fluorescent trajectory does not traverse full channel length within field of view) particle trajectories were removed from the stack by clicking on “Delete Image Slice” button. Once all vacant micrographs were removed, the gray-scale contrast was enhanced using one of the contrast adjustment methods (Fig. S2). The transform was saved, then the background was removed with optimal “Gaussian Standard Deviation” and “Approx. Feature Size” values were identified through the rapid transform process. The transformed image was then exported in the native file type of the original image (e.g., 16-bit TIFF). The channel edge coordinates were then entered into the “X-Y Channel Edge Positions [px]” and saved by selecting “Use Discrete Channel Edge Locations” (Fig. S1). For calculating the acoustic contrast of particles or calculating the acoustic energy density, the “Acoustic Analysis” tab or “Energy Density Cal.” tabs were selected, respectively. The transformed image was loaded into the active window and channel edges were displayed by selecting “Load Transformed Image” and “Display Channel Edge”, respectively. Known values were entered into their corresponding sections. Once the correct values were entered, the fluorescent trajectory was traced by selecting “Trace Particle Trajectory.” The trajectory was traced from the binarized image generated via Otsu thresholding [3]. Adjoining foreground pixels were classified as a foreground object and the object must traverse the entire field of view to be classified as a valid MP trajectory (i.e., contain x-coordinates spanning width of camera sensor). Once a foreground object was classified as a valid trajectory, a best fit curve of the transverse centerline was calculated. Elapsed time, longitudinal position, and transverse position data were then calculated and used to calculate the acoustic contrast factor (Φ) by selecting “Calculate Acoustic Contrast Factor.” Similarly, when calculating the acoustic energy density, the “Calculate Acoustic Energy Density” button was selected. The traced trajectory data, which contains elapsed time, longitudinal location, and transverse position data at pixel-level resolution, was exported by selecting “Export Trajectory Data.” User specified input variables that dictated the fit of acoustic contrast or energy density were then exported by selecting “Export Acoustic Contrast Fit Data” or “Export Energy Density Fit Data,” respectively (Fig. S3). Lastly, once all images were analyzed and data exported, the “Format Files” tab was selected. Acoustic contrast or energy density data was then concatenated to common spreadsheets that were divided by the user specified “File Division Group” (Fig. S4).

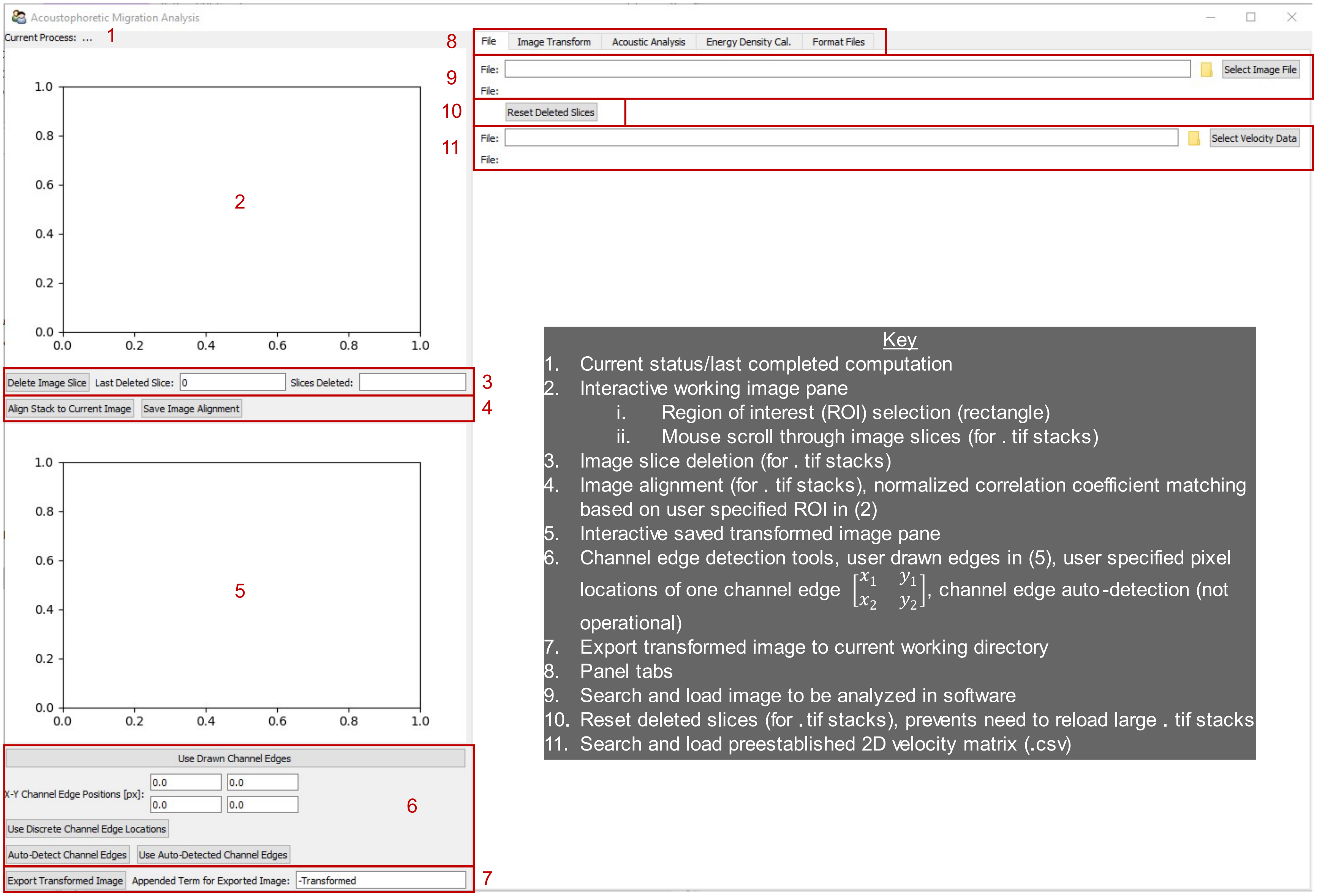

Fig. S1. “File” tab of Python-based GUI software, which is the default view upon initialization of the software.

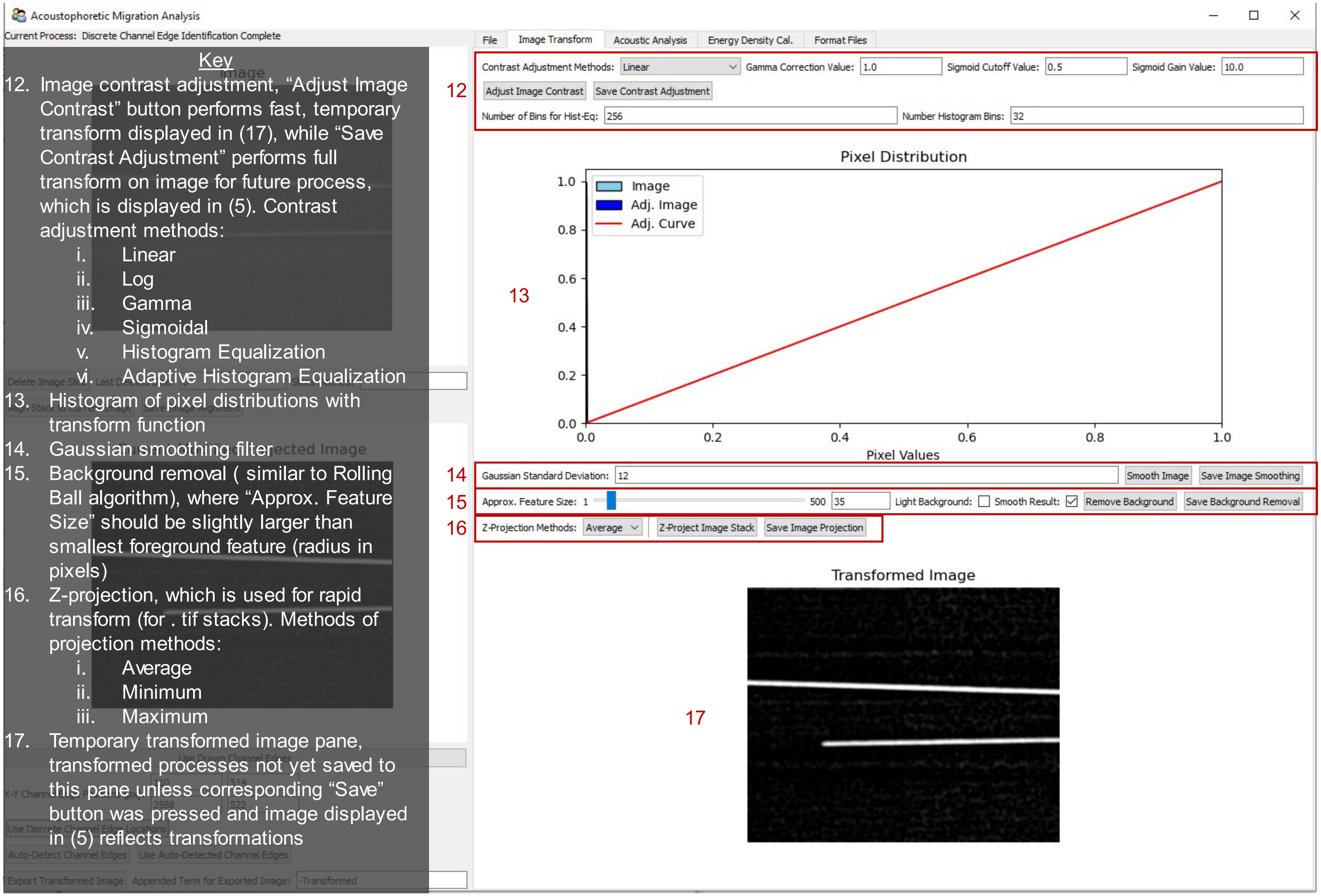

Fig. S2. "Image Transform" tab of Python-based GUI software. Tab used for image enhancement and background removal for more accurate fluorescent trajectory tracing.

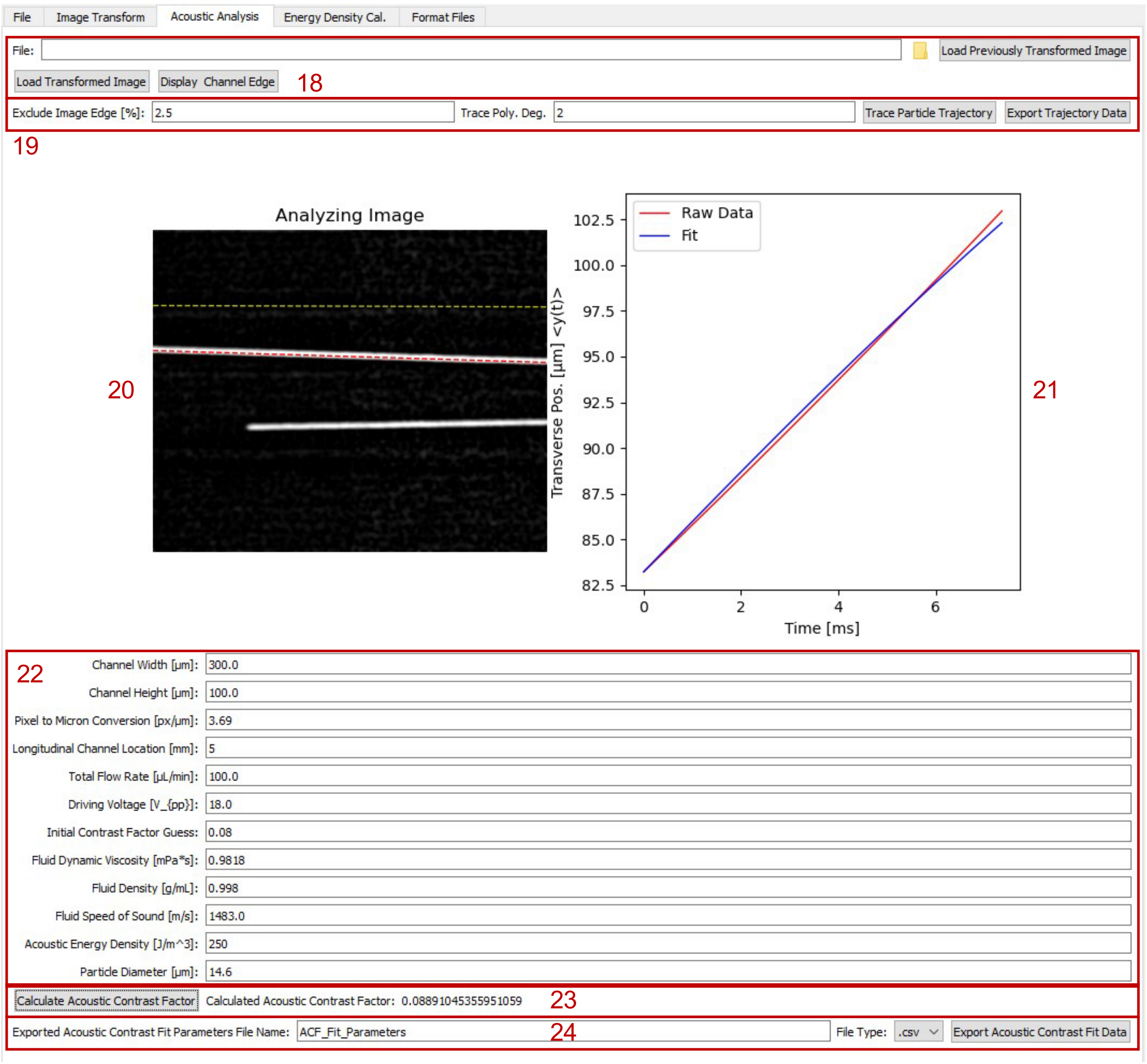

Key

18. Search and load already transformed image for trajectory analysis
19. Trajectory tracing, with edge exclusion input and degree polynomial fitting, and export of trajectory data (time, longitudinal position, transverse position)
20. Image being analyzed, yellow dashed lines indicate channel edge, red and blue lines are traced trajectories
21. Traced trajectory closest to channel edge (red line), with fitted trajectory for best fit value of Φ
22. User input values for calculating best fit value of Φ, hover mouse over corresponding input region for description of input value
23. Best fit calculated value of Φ using user specified values in (22)
24. Exporting best fit calculated value of Φ and associated user specified values in (22)

Fig. S3. "Acoustic Analysis" tab of Python-based GUI software. Tab used for calculating the acoustic contrast of MPs. "Energy Density Cal." tab is similar in appearance and function, but used for calculating the acoustic energy density during device calibration.

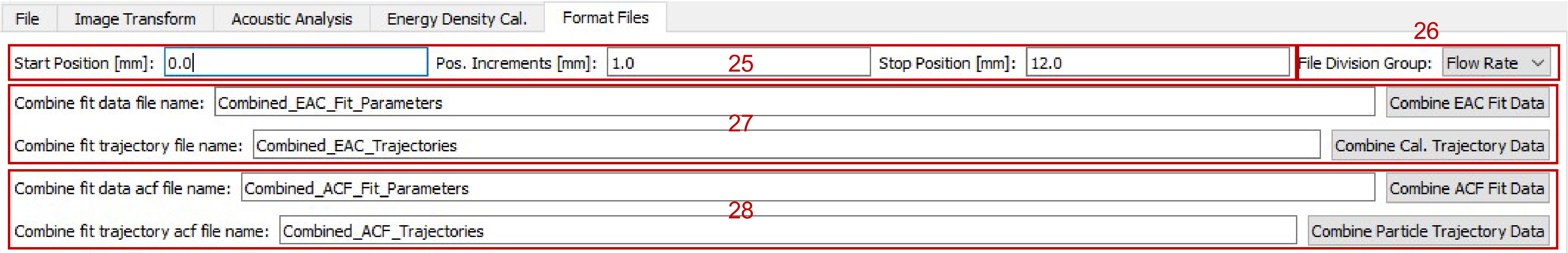

Key

25. Input for organizing data output based on longitudinal position
26. User specific file division, previously exported .csv's (fit and trajectory outputs) will be concatenated to a common .xlsx f ile specified in input bars in (27) and/or (28), but separate files will be created depending on division group with value of division group a ppended to each common file (e.g., for "Flow Rate" division, exported files: "Combined_EAC_Fit_Parameters_100.0uL -min.xlsx" with the voltages separated by sheets)
27. Concatenation of acoustic energy density data
28. Concatenation of acoustic contrast data

Fig. S4. "Format Files" tab of Python-based GUI software. This tab is used to concatenate exported csv's that contain either trajectory tracing data or calculated values with their corresponding fit parameters.

S3. Acoustic energy density along chip

For each experiment, acoustic energy density was calculated during the calibration steps, prior to measuring the acoustic contrast of the polyacrylamide MPs, as described in the main manuscript in the Microparticle acoustophoretic characterization section. We observed a longitudinal dependence on the calculated acoustic energy density, where the energy density monotonically increased along the length of the channel over the region measured, with the exception of the 24 V condition at 6 mm (Fig. S5). This position-dependent energy density was taken into account in the acoustic contrast calculations.

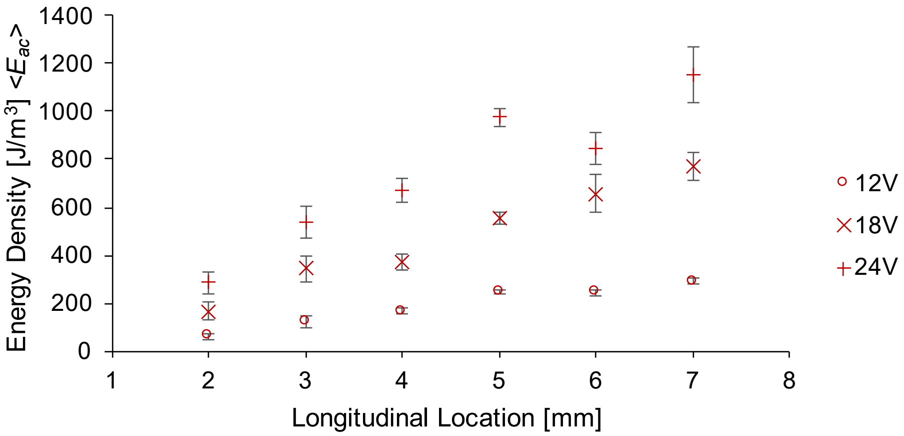

Fig. S5. Calculated acoustic energy density (E_{ac}) for the three voltages tested in this study at the six different longitudinal locations.

S4. Analysis of operational parameters effect on calculated acoustic contrast

To assess systematic bias across the operating parameters, we performed a correlation analysis between the calculated acoustic contrast and the corresponding operating parameters used for each MP lot (Fig. S6). If the *p*-value in the Pearson correlation calculation was greater than 0.01, the operating parameter was considered uncorrelated and did not statistically contribute to the calculated acoustic contrast.

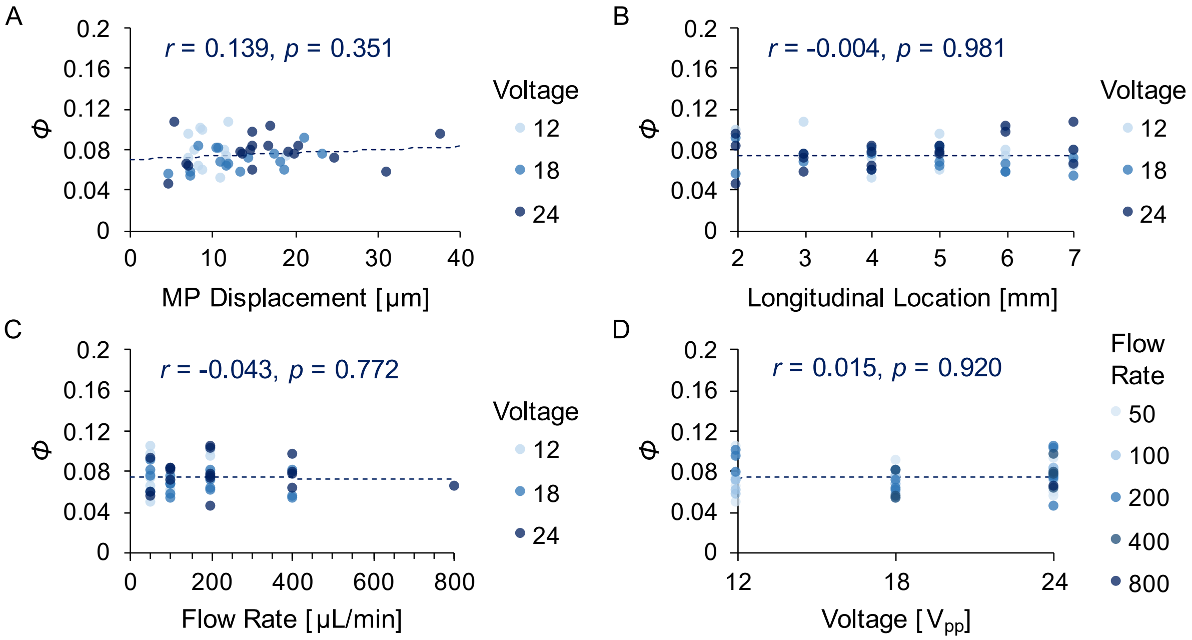

Fig. S6. Correlation plots for polyacrylamide microparticle lot MP-3. "r" is the Pearson correlation coefficient and "p" is the corresponding p-value.

S5. Identification of multiple populations

Common statistical tests were used to assess whether the transverse position data were interpreted as a single population or as two populations, where a high-level overview is outlined in Fig. S7. First, the shape of the transverse position distribution was tested using a Tukey Lambda probability plot correlation coefficient (PPCC), where the shape parameter value corresponding to the maximum of the correlation coefficient provides insight into which type of distribution might best match the data. Common values are shown in Fig. S8. While this is not a definitive test, it provides insight into potential distributions that would model the data more appropriately and assumes symmetry. For example, if the transverse position data was best represented by a normal distribution (i.e., shape parameter, λ, was approximately 0.14), the population was unlikely to contain multiple subpopulations. Alternatively, a best fit shape parameter that is greater than 0.14 suggests a short-tailed distribution, and values approaching 0.5 suggest a U-shaped distribution (Fig. S8A).

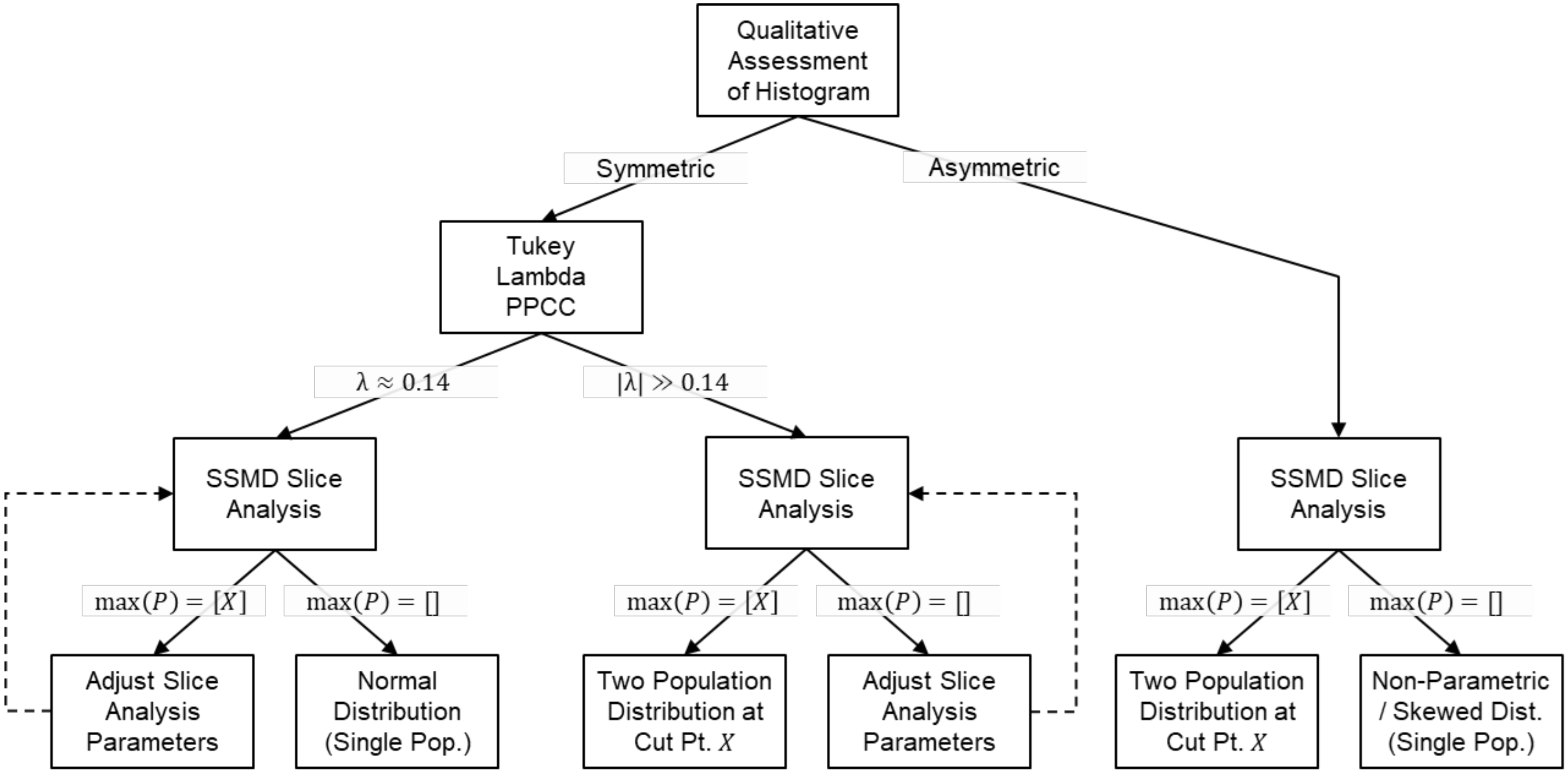

Fig. S7. High-level decision tree for assessing which distribution more appropriately fits transverse position data. SSMD stands for strictly standardized mean difference and variables λ, P, and X correspond to the best fit Tukey Lambda PPCC shape parameter, prominence, and peak prominence of strictly standardized mean difference. Empty brackets "[]" when calculating peak prominence suggest lack of optimal cut point to separate the distribution into two sub-populations.

Following the Tukey Lambda PPCC, we then tested whether the transverse position data could be separated into two distributions and at what optimal value. To do this, the transverse position data were sorted and systematically separated into two distributions, with a group that that was equal to or lower than a cut value and a second group that was greater than a cut value. The cut value was incrementally increased starting at the 1st percentile to the 99th percentile of the data. The strictly standardized mean difference between the two separated distributions was calculated at each cut value. The strictly standardized mean difference was plotted with respect to the cut value, and the cut value that resulted in the maximum prominence along the curve was taken as the optimal value to separate the two subpopulations.

Results from the PPCC analysis suggested the distributions of the transverse position data for Donors 1 and 2 were unlikely normally distributed and therefore merited analysis as two populations. Additionally, the distribution appeared asymmetric for Donor 2 given the greater percentage of cells exhibiting high migration, which limited to applicability of the PPCC test. Confirming this, distinct maximum prominence points were present in the strictly standardized mean difference curve for Donors 1 and 2. In contrast, Donor 3 exhibited no cut value having significant prominence supporting interpretation as a unimodal population (Fig. S8B).

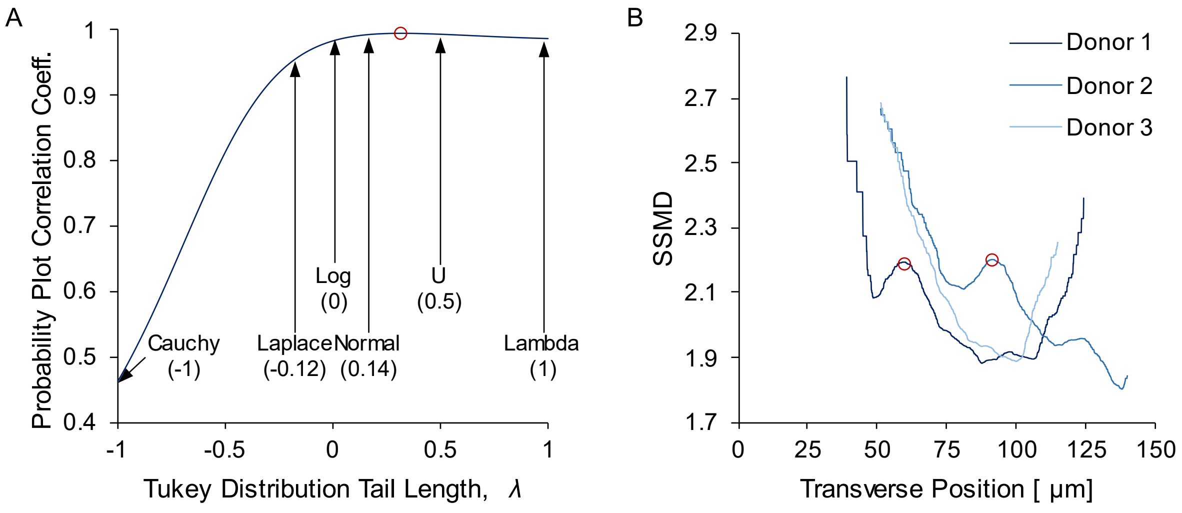

Fig. S8. A) Tukey Lambda PPCC for Donor 2 transverse position data. Common distributions with corresponding quantile value are shown for reference. Red circle indicates max correlation value. B) Calculated strictly standardized mean difference (SSMD) when separating transverse position data into two distributions at the corresponding transverse position cut point. Red circles "○" correspond to cut points resulting in maximum prominence.

S6. References

[1] R. Dubay, J. Fiering and E. M. Darling, "Effect of elastic modulus on inertial displacement of cell-like particles in microchannels," *Biomicrofluidics,* vol. 14, no. 4, p. 044110, 2020.

[2] N. A. Mortensen, F. Okkels and H. Bruus, "Reexamination of Hagen-Poiseuille flow: shape-dependence of the hydraulic resistance in microchannels," *Physical review E: Statistical, nonlinear, and soft matter physics,* vol. 71, no. 5, p. 057301, 2005.

[3] N. Otsu, "A Threshold Selection Method from Gray-Level Histograms," *IEEE Transactions on Systems, Man, and Cybernetics,* vol. 9, no. 1, pp. 62-66, 1979.
